# Identification of unique transcriptomic signatures and key genes through RNA sequencing and integrated WGCNA and PPI network analysis in HIV infected lung cancer

**DOI:** 10.1002/cam4.4853

**Published:** 2022-05-24

**Authors:** Liwei Wu, Yongfang Chen, Laiyi Wan, Zilu Wen, Rong Liu, Leilei Li, Yanzheng Song, Lin Wang

**Affiliations:** ^1^ Department of Thoracic Surgery Shanghai Public Health Clinical Center, Fudan University Shanghai Shanghai China; ^2^ Department of Pharmacy Shanghai Public Health Clinical Center Shanghai China; ^3^ Department of Scientific Research Shanghai Public Health Clinical Center, Fudan University Shanghai China; ^4^ TB Center Shanghai Emerging and Re‐emerging Infectious Disease Institute, Fudan University Shanghai China

**Keywords:** differential gene analysis, HIV infected lung cancer, hub gene, PPI, WCGNA

## Abstract

With the widespread use of highly active antiretroviral therapy (HARRT), the survival time of AIDS patients has been greatly extended. However, the incidence of lung cancer in HIV‐infected patients is increasing and has become a major problem threatening the survival of AIDS patients. The aim of this study is to use Weighted Gene Co‐expression Network Analysis (WGCNA) and differential gene analysis to find possible key genes involved in HIV‐infected lung cancer. In this study, using lung tissue samples from five pairs of HIV‐infected lung cancer patients, second‐generation sequencing was performed and transcriptomic data were obtained. A total of 132 HIV‐infected lung cancer‐related genes were screened out by WGCNA and differential gene expression analysis methods. Based on gene annotation analysis, these genes were mainly enriched in mitosis‐related functions and pathways. In addition, in protein–protein interaction (PPI) analysis, a total of 39 hub genes were identified. Among them, five genes (ASPM, CDCA8, CENPF, CEP55, and PLK1) were present in both three hub gene lists (intersection gene, DEGs, and WCGNA module) suggesting that these five genes may become key genes involved in HIV‐infected lung cancer.

## Graphical abstract

1. This study reveals a unique transcriptomic profile of HIV‐infected lung cancer patients. 2.This study could point the way to studying the pathogenesis of lung cancer in HIV infection.

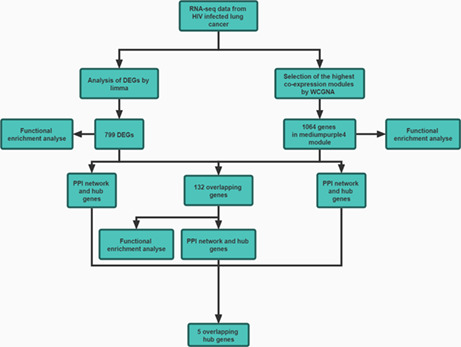

## 1 INTRODUCTION

Lung cancer is one of the most common types of cancer in the world.^1^ In recent years, with the use of highly active antiretroviral therapy (HARRT), the survival time of HIV patients has been increasing.^2^ Among HIV patients, the prevalence of lung cancer has also been increasing.^3^, ^4^ According to the Global Cancer Statistics report, lung cancer has the third highest mortality rate among all malignancies.^5^ And the survival rate of HIV‐infected lung cancer patients is lower than that of regular lung cancer patients.^4^ According to National Comprehensive Cancer Network (NCCN) guidelines, the treatment of lung cancer can be divided into the following categories: Surgery, chemotherapy, radiotherapy, targeted drug therapy, and immunotherapy.^6^ For HIV‐infected lung cancer patients, the survival advantage for patients is extremely limited, despite the variety of treatment options.^7^ Therefore, it is important to explore specific mechanisms of lung cancer in HIV infection and biomarkers for early diagnosis.

With the development of transcriptome sequencing technology, more tumor‐related genes and mechanisms have been discovered.^8^ Among them, advances in differential gene analysis have spawned a boom in the field of tumor therapy and diagnosis.^9^, ^10^ The Weighted Gene Co‐expression Network Analysis (WGCNA) is an important approach to understand the relationship between genes and clinical phenotypes.^11^, ^12^ Through WGCNA analysis, gene modules that are closely associated with the clinical phenotype of interest can be identified. These gene modules contain genes that correlate with selected clinical phenotypes.^13^ The combination of WGCNA and differential analysis allows the full range of genes associated with HIV‐infected lung cancer to be obtained. Subsequent screening of hub genes could yield gene targets and biomarkers that could be potential therapeutic targets for HIV‐infected lung cancer.

In this study, the mRNA expression data of HIV‐infected lung cancer from the Shanghai Public Health Clinical Center were analyzed by WGCNA and differential gene expression analysis to obtain HIV‐infected lung cancer‐related genes. We further identified genes that could be potential targets for treatment and diagnose through protein–protein interaction and functional enrichment analysis. This study provides a potential basis for the treatment of HIV‐infected lung cancer patients and points the way to new drug development.

## 2 MATERIALS AND METHODS

### 2.1 Patient cohorts and transcriptome data

We collected five tumor samples and five normal control samples from five HIV‐infected lung cancer patients. Among them, three patients were diagnosed with adenocarcinoma and two patients were diagnosed with squamous carcinoma. And in the preoperative staging, two patients were determined to be Stage IA, two patients were determined to be Stage IB, and one patient was determined to be Stage IIB. Tumor samples and normal control samples were frozen at −20 and subsequently stored at −80. Total RNA of tissue samples was extracted using the standard RNA extraction method with TRIzol (Invitrogen). Tissue samples were sequenced by 50 bp single‐end sequencing on an Illumina HiSeq 2500 sequencer, and were annotated to a reference transcript set of Human hg38 gene standard track. According to edgeR help file, genes with low read counts could not usually use to do further analysis. So, we kept the genes with a cpm (count per million) ≥1 in this study.^14^ After filtering using function rpkm in edgeR package, which is calculated by dividing gene counts by gene length, a total of 16,875 genes with RPKM values were subject to our next analysis. Informed consent and an ethical approval (Shanghai Public Health Clinical Center Ethical Committee) were obtained.

### 2.2 Key co‐expression WGCNA modules

The methods of co‐expression networks are used to screen candidate biomarkers and therapeutic targets based on network calculation. In our study, the gene expression data matrix of HIV‐infected lung cancer was constructed to gene co‐expression networks using the R package WGCNA.^15^ WGCNA can be used to divide the gene expression matrix into several modules, each containing a certain number of genes that are closely associated with a selected clinical phenotype. In our study, two phenotypes, tumor and normal, were selected. The specific WGCNA analysis parameters in this study were set as follows: Soft powers *β* = 10 was selected using the function pickSoftThreshold; WGCNA 'mergeCutHeight' was set as 0.45. Then, the adjacency matrix was created by the following formula:

$$a_{\mathrm{ij}}=\left|S_{\mathrm{ij}}\right|\beta$$

a_ij_: adjacency matrix between gene i and gene j; S_ij_: similarity matrix which is done by Pearson correlation of all gene pairs; *β*: softpower value.

The adjacency matrix was transformed into a topological overlap matrix (TOM) as well as the corresponding dissimilarity (1‐TOM). Afterwards, a hierarchical clustering dendrogram of the 1‐TOM matrix was constructed to classify the similar gene expressions into different gene co‐expression modules. To further identify the modules associated with clinical phenotypes, the relationship between clinical phenotypes and modules was calculated. Therefore, modules with high correlation coefficients were considered candidates relevant to clinical phenotypes and were selected for subsequent analysis.

### 2.3 Differential expression analysis

The R package limma allows easy processing of the gene expression matrix to obtain differentially expressed genes (DEGs) between the two groups.^16^ We used the limma R package to obtain DEGs between HIV‐infected lung cancer samples and HIV‐infected normal samples. The p‐value was adjusted by the Benjamini–Hochberg method to control for the false discovery rate (FDR). Genes with the cutoff criteria of |logFC| ≥ 1.0 and adj. *p* < 0.05 were regarded as DEGs. The DEGs of the gene expression matrix were visualized as a volcano plot and heatmap by using the R package ggplot2 and pheatmap.^17^, ^18^ Subsequently, genes in the modules of co‐expression network most associated with the clinical phenotype and the DEGs were subsequently used as intersection genes, which were presented as a Venn diagram using the R package VennDiagram.^19^

### 2.4 Functional annotation

To explore Gene Ontology (GO) and Kyoto Encyclopedia of Genes and Genomes (KEGG) of DEGs, WGCNA module genes, and intersection genes, R package clusterProfiler package was used to explore the functions and pathways, with a cut‐off criterion of adjusted *p* < 0.05.^20^ GO annotation that contains the three sub‐ontologies—biological process (BP), cellular component (CC), and molecular function (MF)—can identify the biological properties of genes and gene sets for all organisms.^21^ KEGG annotation can identify the pathways of DEGs, WGCNA module genes, and intersection genes.^22^

### 2.5 Construction of PPI and screening of hub genes

The STRING database is a tool that has been used to predict protein–protein interactions (PPI).^23^ In this study, we used the STRING database to construct PPI networks for DEGs, WGCNA module genes, and intersection genes. The constructed PPI networks were then imported into cytoscape software (version: 3.8.2) to screen hub genes using the cytoHubba plugin.^24^, ^25^ We screened the hub genes using the Maximal Clique Centrality (MCC) algorithm, and the top 10 or 20 genes were ranked in descending order according to the MCC scores.^26^ In this study, the genes with the top 10 or 20 MCC scores were considered as hub genes. We then took the intersection of hub genes from the three gene lists (DEGs, WGCNA module genes, and intersection genes) as candidate biomarkers, visualizing by venn diagram.^19^

## 3 RESULTS

**FIGURE 1:**
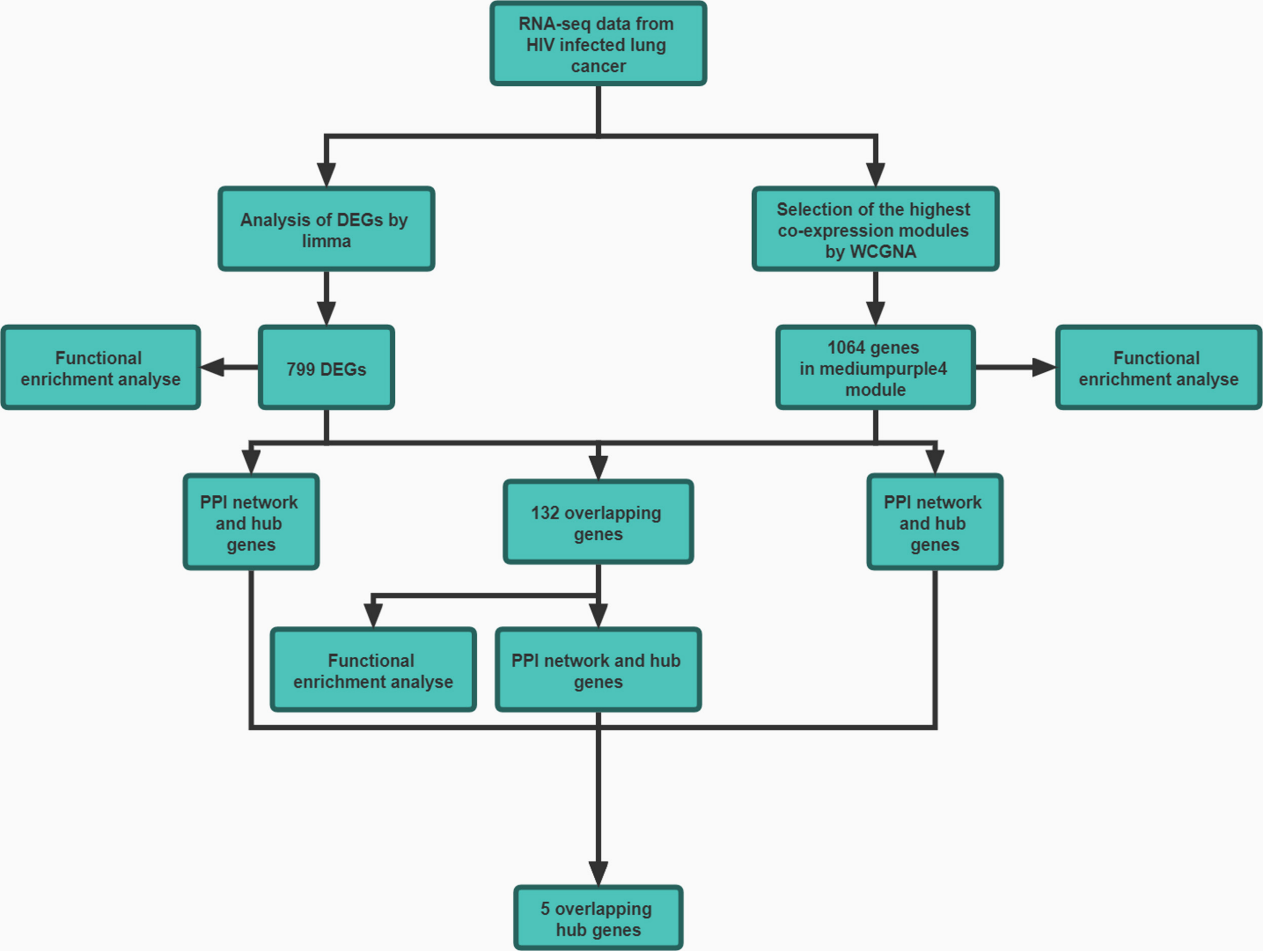
The work flow of this study

The workflow of this study was presented as Figure 1.

### 3.1 Construction of weighted gene co‐expression modules

**FIGURE 2:**
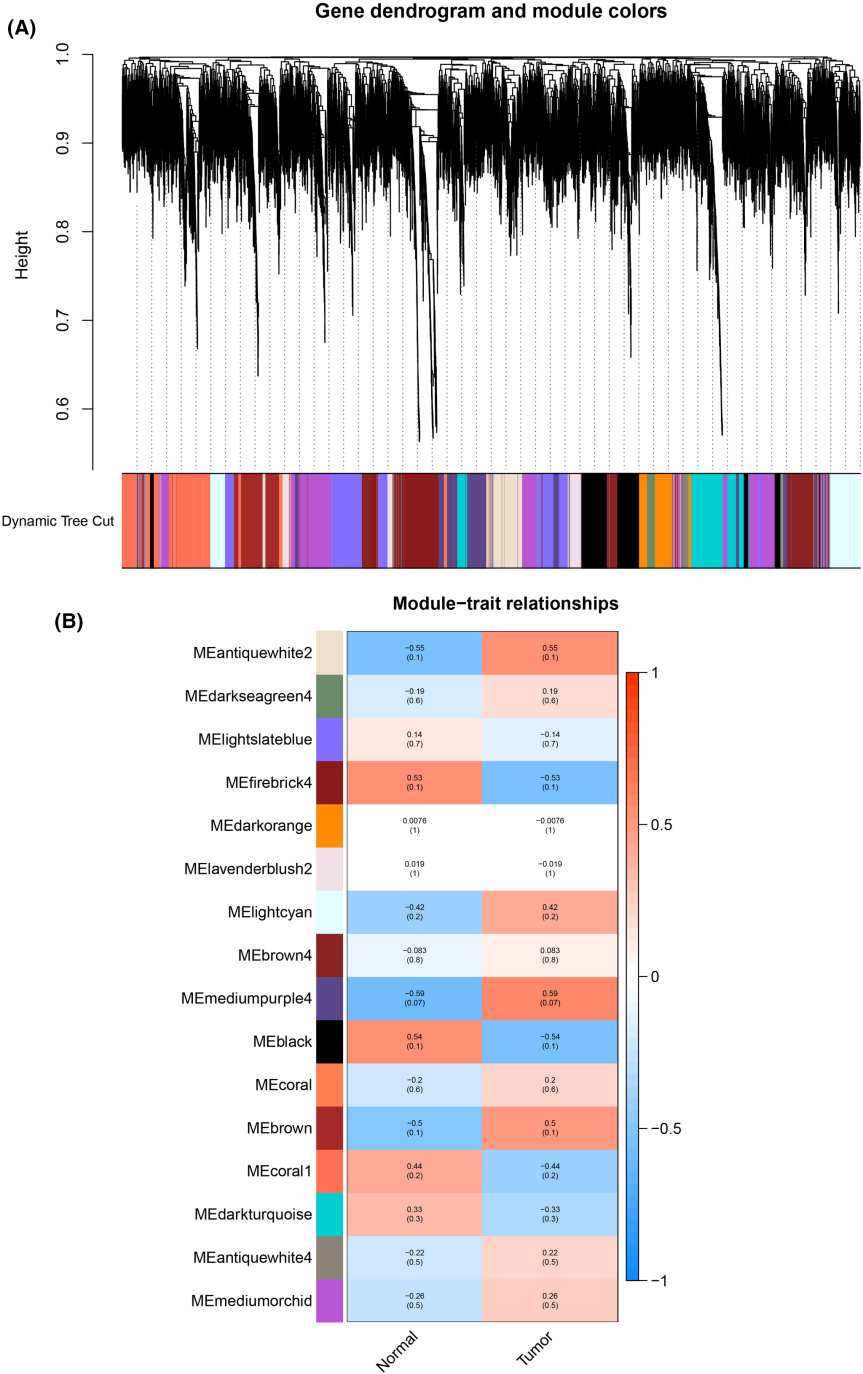
The results of WGCNA analysis, identifying modules associated with the clinical phenotypes. (A) The cluster dendrogram of co‐expression network modules was ordered by a height value of genes based on the 1‐TOM matrix. Each module was assigned different colors. (B) Module‐phenotypes relationships. Each row corresponds to a color module and column corresponds to a clinical phenotype (cancer and normal). Each module contains the corresponding correlation value and *p*‐value.

To explore genes closely associated with the clinical phenotype, we used the WGCNA R package to conduct analysis for the gene expression matrix. Each module was assigned a different color, and a total of 16 modules were identified (Figure 2A). We then plotted a heatmap of module‐phenotype relationships to assess the association between each module and two clinical phenotypes (cancer and normal). The results of the module‐ phenotype relationships were presented in Figure 2B, revealing that the mediumpurple4 module was found to have the highest association with tumor tissues (*r* = 0.59, *p* = 0.07).

### 3.2 Identification of DEGs

**FIGURE 3:**
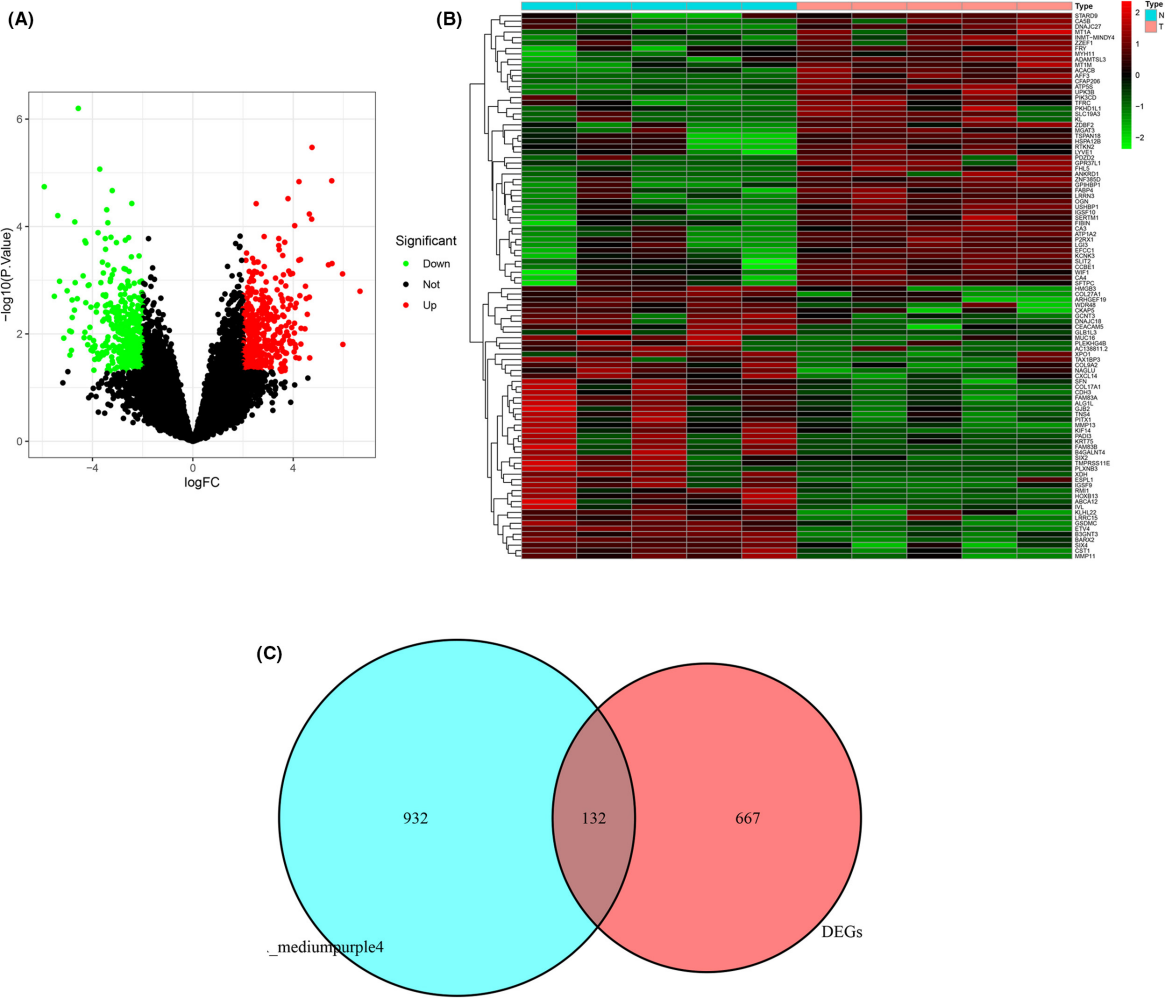
Identification of differentially expressed genes (DEGs) of HIV infected lung cancer. (A) The volcano plot of DEGs. (B) The heatmap of DEGs. (C) The venn diagram of mediumpurple4 module and DEGs.

Based on the screening criteria of |logFC| ≥ 1.0 and adj. *p* < 0.05, a total of 799 DEGs were found to be dysregulated in tumor tissues by the limma package (Figure 3A,B). Among them, 395 genes were up‐regulated and 404 genes were down‐regulated. A total of 1064 genes were found in mediumpurple4 module. As shown in Figure 3C, 132 intersection genes were found between DEGs and mediumpurple4 module.

### 3.3 Construction of PPI network

**FIGURE 4:**
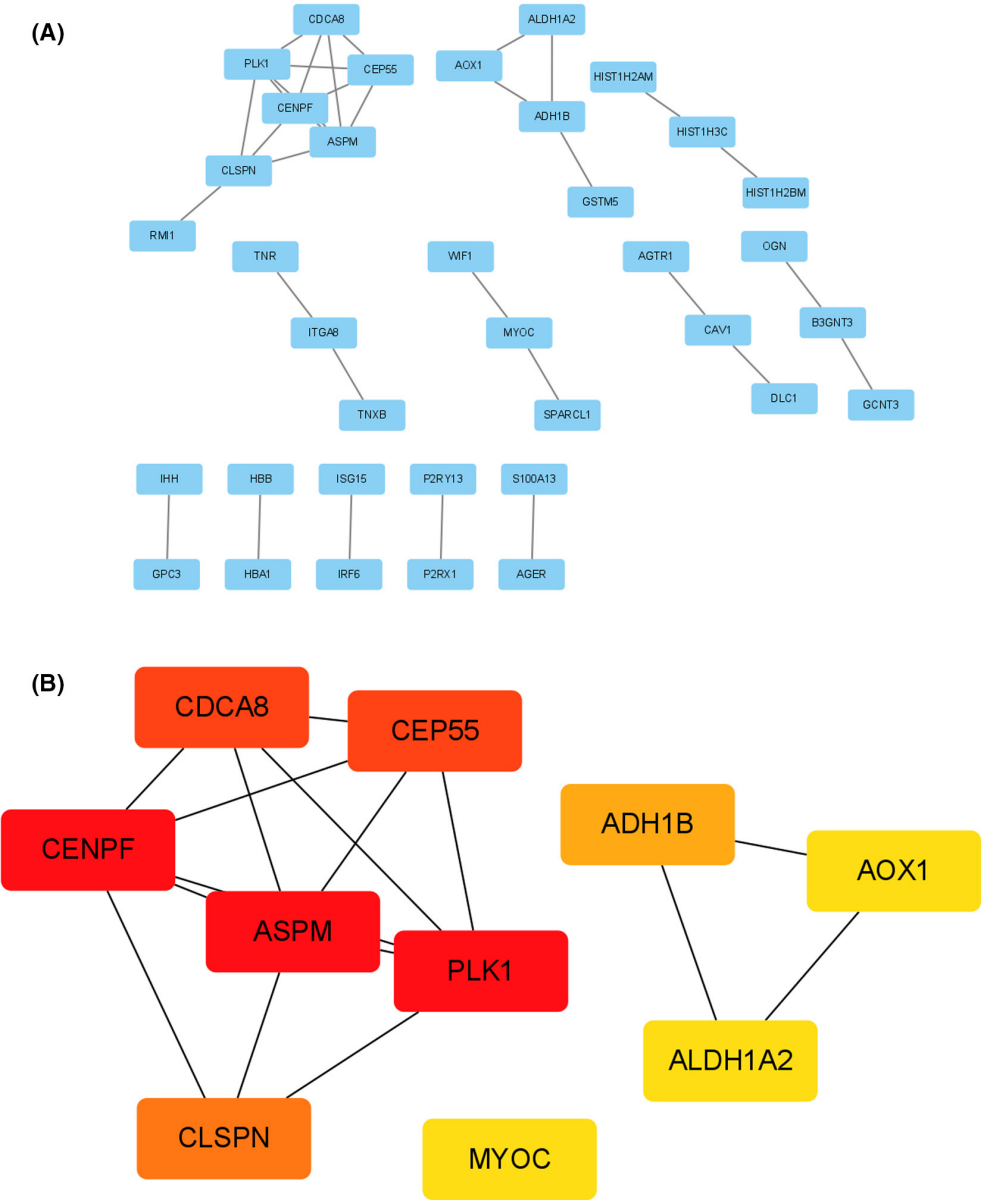
Visualization of the protein–protein interaction (PPI) network and the candidate hub genes of 132 overlapping genes. (A) PPI network of 132 overlapping genes. (B) Hub genes of 132 overlapping genes. Darker red of nodes presents higher MCC score.

**FIGURE 5:**
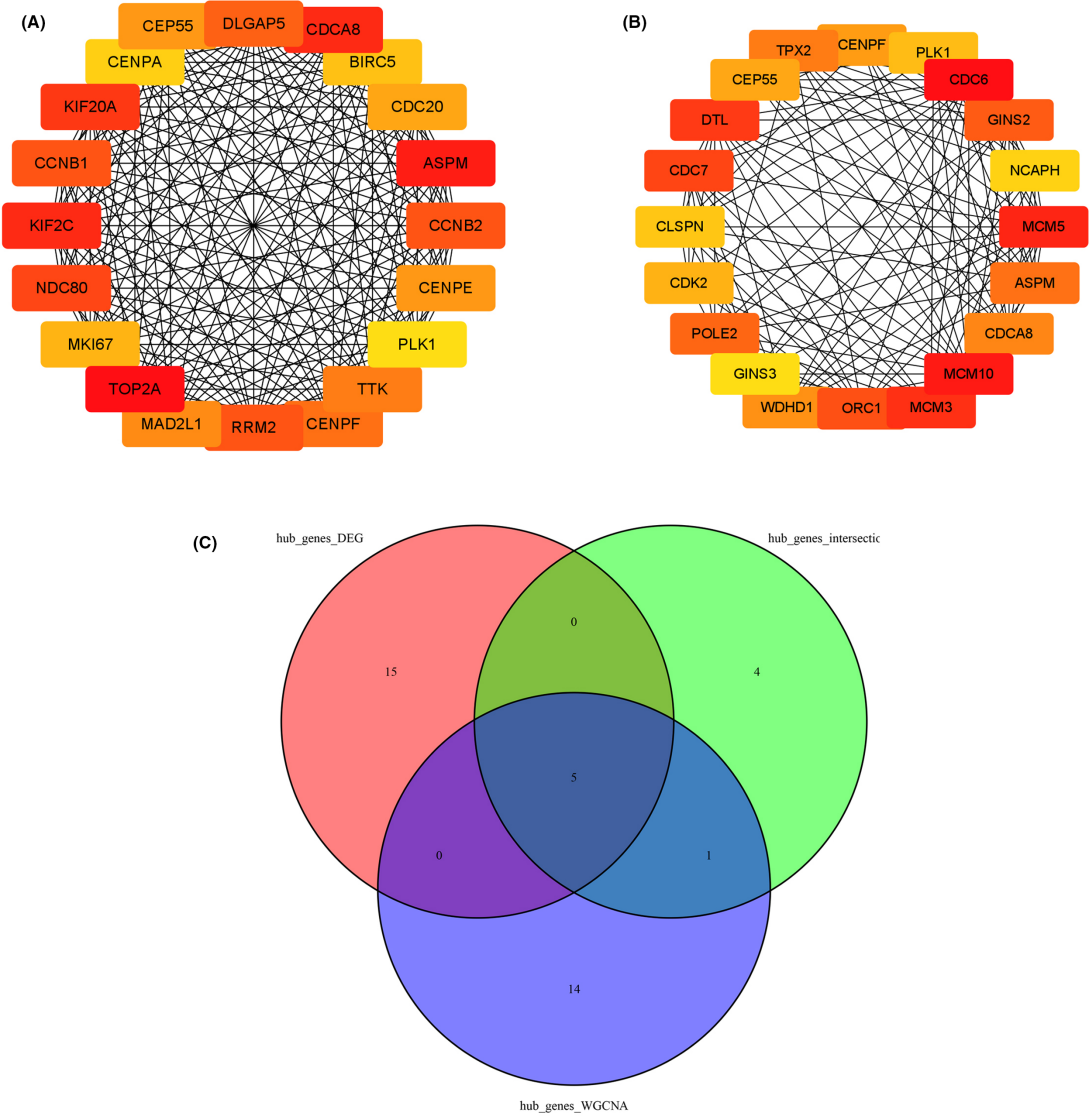
Visualization of hub genes of DEGs and mediumpurple4 module. (A) Hub genes of DEGs. (B) Hub genes of mediumpurple4 module. (C) The intersection among hub genes of 132 overlapping genes, DEGs, and mediumpurple4 module.

We constructed the PPI network of intersection genes using the STRING database, and based on the screening conditions of minimum required interaction score >0.7 (Figure 4A). The hub genes selected from the PPI network using the MCC algorithm of CytoHubba plugin were shown in Figure 4B. According to MCC scores, the top 10 highest‐scored genes were selected as hub genes. Subsequently, we constructed the PPI network of DEGs and mediumpurple4 module, screening hub genes by MCC algorithm of CytoHubba plugin. According to MCC scores, the top 20 highest‐scored genes were selected as hub genes (Figure 5A and Figure 5B). The intersection of three gene lists (intersection hub genes, DEGs hub genes and mediumpurple4 module hub genes), including ASPM, CDCA8, CENPF, CEP55, and PLK1, was shown as Figure 5C. The detailed MCC scores were shown as Table 1.

**TABLE 1:** MCC rank of three gene lists

Rank	Name	Score
Intersection genes MCC rank		
1	ASPM	30
1	PLK1	30
1	CENPF	30
4	CEP55	24
4	CDCA8	24
6	CLSPN	7
7	ADH1B	3
8	MYOC	2
8	ALDH1A2	2
8	AOX1	2
DEGs MCC rank		
1	TOP2A	1.8941E+24
2	ASPM	1.8941E+24
3	KIF2C	1.8941E+24
3	CDCA8	1.8941E+24
5	KIF20A	1.8941E+24
6	NDC80	1.8941E+24
7	CCNB1	1.8941E+24
7	RRM2	1.8941E+24
7	CCNB2	1.8941E+24
10	DLGAP5	1.8941E+24
11	CENPF	1.89409E+24
12	TTK	1.89409E+24
13	MAD2L1	1.89409E+24
14	CENPE	1.89399E+24
14	CEP55	1.89399E+24
16	CDC20	1.89297E+24
17	MKI67	1.89297E+24
18	BIRC5	1.8918E+24
19	CENPA	1.8918E+24
20	PLK1	1.89067E+24
Mediumpurple4 module MCC rank		
1	CDC6	413,198
2	MCM10	364,200
3	MCM5	291,870
4	MCM3	264,970
5	DTL	230,170
6	CDC7	208,801
7	ORC1	208,236
8	GINS2	205,560
9	POLE2	188,051
10	ASPM	159,367
11	TPX2	153,749
12	CDCA8	150,016
13	WDHD1	145,202
14	CENPF	133,086
15	CEP55	132,720
16	CDK2	120,978
17	PLK1	94,091
18	CLSPN	82,568
19	NCAPH	52,104
20	GINS3	45,360

### 3.4 Functional enrichment analyses for three gene lists

**FIGURE 6:**
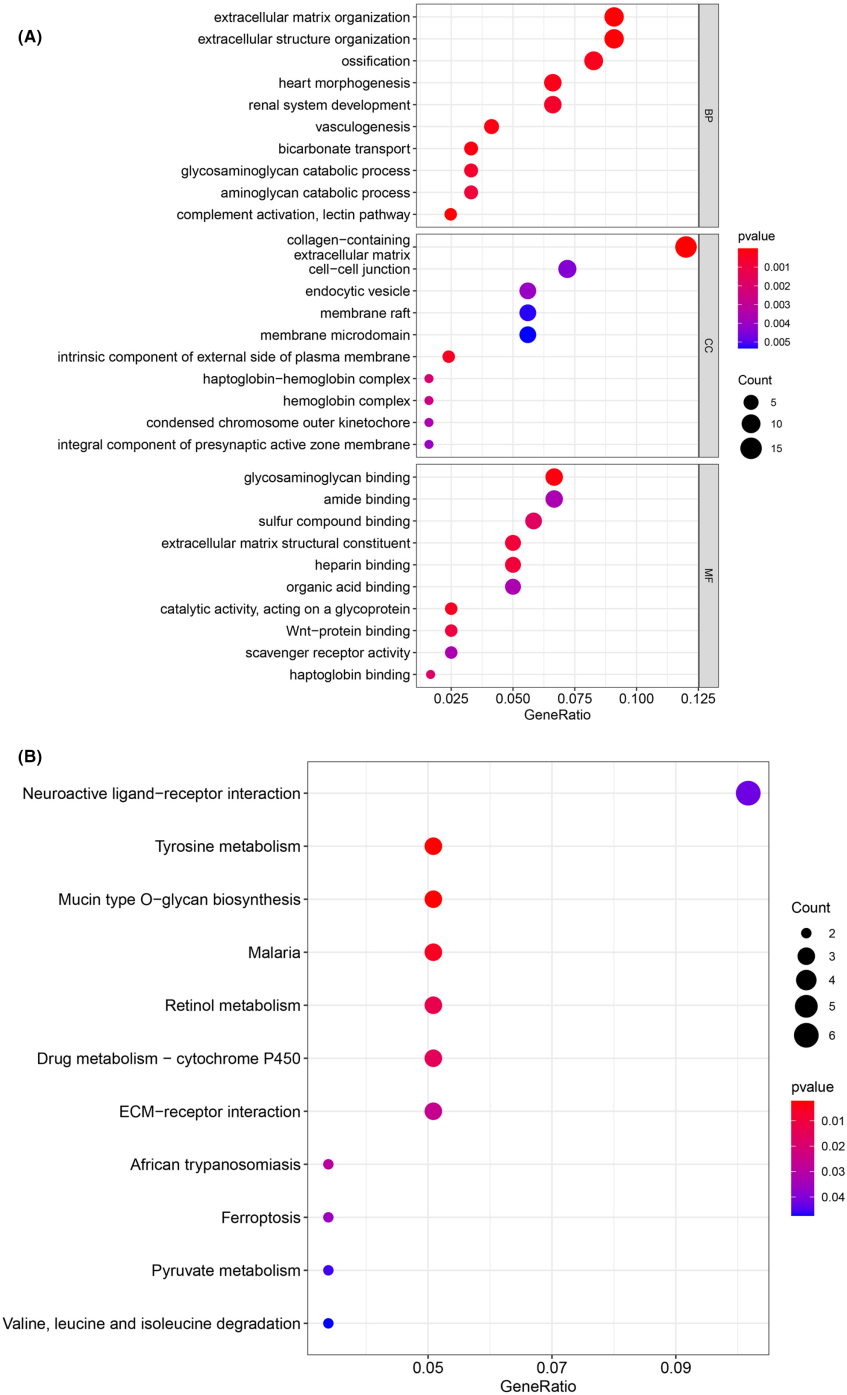
The enrichment analysis. The red dots present low P value, while blue dots present high *p* value. (A) GO annotation of 132 overlapping genes. (B) KEGG pathways of 132 overlapping genes.

In GO enrichment analysis of intersection genes, we observed several enriched gene sets shown in Figure 6A. The biological process (BP) was mainly enriched in extracellular matrix organization, extracellular structure organization and vasculogenesis. For the result of the cellular component (CC), it was revealed that these genes were mainly involved in collagen−containing extracellular matrix, intrinsic component of external side of plasma membrane and haptoglobin−hemoglobin complex. Moreover, in the molecular function (MF) analysis, glycosaminoglycan binding, catalytic activity, acting on a glycoprotein, and extracellular matrix structural constituent were indicated to be related to the intersection genes. In KEGG enrichment analysis (Figure 6B), the intersection genes were mainly enriched in Tyrosine metabolism, Mucin type O—glycan biosynthesis, Malaria, Retinol metabolism, Drug metabolism—cytochrome P450, and ECM‐receptor interaction. The GO and KEGG enrichment analysis of DEGs and mediumpurple4 module were shown as Table 2 and Table 3.

**TABLE 2:** Top 20 GO annotation of DEGs

Category	Description	Count	Log10(P)
GO Cellular Components	External encapsulating structure	59	−18.5
GO Biological Processes	Head development	62	−13.54
GO Biological Processes	Blood vessel development	60	−13.25
GO Biological Processes	Embryonic morphogenesis	51	−12.9
GO Biological Processes	Circulatory system process	49	−11.59
GO Biological Processes	Actin filament‐based process	58	−11.43
GO Biological Processes	Tissue morphogenesis	49	−11.18
GO Biological Processes	Ossification	38	−10.7
GO Cellular Components	Cell–cell junction	42	−10.52
GO Biological Processes	Skeletal system development	42	−10.16
GO Biological Processes	Ion homeostasis	55	−10.16
GO Biological Processes	Epithelial cell proliferation	39	−10.11
GO Biological Processes	Mitotic cell cycle process	51	−9.61
GO Biological Processes	Chemotaxis	45	−9.3
GO Molecular Functions	Glycosaminoglycan binding	26	−9.24
GO Biological Processes	Negative regulation of cell differentiation	48	−9.08
GO Biological Processes	Muscle structure development	46	−9.08
GO Biological Processes	Regulation of growth	46	−8.6
GO Molecular Functions	Calcium ion binding	48	−8.48
GO Biological Processes	Cell junction organization	47	−8.36

**TABLE 3:** Top 20 GO and KEGG annotation of mediumpurple4 module

Category	Description	Count	Log10(P)
GO Biological Processes	Cellular response to growth factor stimulus	64	−11.82
GO Cellular Components	Focal adhesion	46	−11.05
GO Biological Processes	Regulation of cell adhesion	65	−10.71
GO Biological Processes	Blood vessel development	65	−10.37
GO Cellular Components	Perinuclear region of cytoplasm	63	−10.15
GO Cellular Components	Extracellular matrix	53	−9.95
GO Biological Processes	Negative regulation of cell population proliferation	64	−9.61
GO Biological Processes	Positive regulation of cellular component biogenesis	49	−9.44
GO Biological Processes	DNA replication	32	−9.08
GO Biological Processes	Chemotaxis	53	−9
KEGG Pathway	Cell adhesion molecules (CAMs)	23	−8.96
GO Biological Processes	Response to inorganic substance	51	−8.91
GO Biological Processes	Ossification	41	−8.78
GO Biological Processes	Cell‐substrate adhesion	38	−8.68
GO Cellular Components	Lytic vacuole	59	−8.66
GO Molecular Functions	Protein domain specific binding	57	−8.42
GO Biological Processes	Regulation of cell morphogenesis	34	−8.41
GO Biological Processes	MAPK cascade	61	−8.37
GO Biological Processes	Epithelial cell differentiation	53	−8.11
GO Biological Processes	Renal system development	33	−8.07

## 4 DISCUSSION

HIV‐infected Lung cancer is the non‐HIV‐specific cancer and there is no evidence that HIV can directly cause lung cancer.^4^ Lung cancer is a malignant tumor that has the third highest mortality rate of all malignancies.^5^ Some studies have shown that lung cancer patients with HIV infection have lower survival rates than patients with regular lung cancer.^4^ According to the NCCN guidelines, early‐stage lung cancer (Stages I and II) can be treated by surgical resection.^6^ However, because of the special immune status of HIV patients, advanced‐stage lung cancer accounts for the majority of cases. With the development of transcriptome sequencing technology, more and more new techniques are being applied to the field of oncology. Differential gene analysis can screen for genes that are differentially expressed in tumor tissues and normal tissues, which are often the main factors driving tumor development. In contrast, WGCNA can construct a weighted network of gene and clinical phenotype correlations, which in turn screens for the gene modules that are most relevant to the clinical phenotype. The combined use of these two approaches allows for the screening of the most critical genes in tumorigenesis and development, and thus the exploration of detailed mechanisms as well as the development of relevant diagnostic biomarkers.

In this study, a total of five genes were identified as possible biomarkers. As suggested in functional and pathway annotation analysis by the clusterProfiler package, these genes were mainly enriched in extracellular matrix and angiogenesis, indicating that most of the genes of the immune system are silent. In the case of ordinary lung cancer, there is usually an activation of immunity to kill tumor cells. The tumor cells will also escape from the immune system. In the case of HIV‐infected lung cancer, there is generally no activation of the immune system, which is consistent with the results of our analysis. Furthermore, according to MCC scores from the CytoHubba plugin in Cytoscape, the top 10 or 20 hub genes of three gene lists were screened out. Among them, ASPM, CDCA8, CENPF, CEP55, and PLK1 were involved in both three gene lists. Finally, we selected above five genes to be biomarkers and key genes for uncovering the mechanisms involved in HIV‐infected lung cancer.

ASPM, also known as assembly factor for spindle microtubules, is essential for normal mitotic spindle function in embryonic neuronal cells. Studies in mice have also shown a role for this gene in mitotic spindle regulation, with a preferential role in the regulation of neurogenesis. Mutations in this gene have been associated with primary type 5 microcephaly.^27^, ^28^ CDCA8, also known as Cell Division Cycle Associated 8, encodes a component of the chromosome passenger complex. This complex is an essential regulator of mitosis and cytokinesis. The protein is regulated by the cell cycle and is required for chromatin‐induced microtubule stabilization and spindle formation.^29^, ^30^ CENP5, also known as Centromere Protein F, encodes a protein that can bind to the centromere‐kinetochore complex. This protein may play a role in chromosome segregation during mitosis. Autoantibodies against this protein can be found in cancer patients. CEP55 plays a role in DNA damage and cytoskeletal signaling.^31^, ^32^ PLK1 belongs to the CDC5/Polo subfamily. It is highly expressed during mitosis and its levels are found to be elevated in many different types of cancer. Inhibition of the expression of this gene in cancer cells can greatly inhibit cancer cell proliferation and may serve as a target for cancer therapy.^33^, ^34^, ^35^ The above five genes are among the important genes that affect mitosis, of which PLK1 and CENP5 have been shown to be associated with cancer. Because of the lack of immune system action, which leads to a large difference between HIV‐infected lung cancer and normal lung cancer at the cellular and tissue level, the expression level of mitosis‐related genes is higher in HIV‐infected lung cancer cells, while the expression level of genes related to targeting the immune system is lower. This is a result of the characteristics of human immunodeficiency virus. Therefore, it is necessary to develop targeted drugs for HIV‐infected lung cancer patients or to expand the existing indications for targeted drugs.

There were several limitations to our study. First, experiments were needed to validate the five hub genes. Secondly, the small number of samples (5 normal samples and 5 lung cancer samples) may affect the accuracy of the analysis results. Finally, more detailed mechanisms need to be verified experimentally. Despite these limitations, we will further investigate the molecular mechanisms of hub genes as well as develop new drugs in future studies.

In summary, through WGCNA and differential gene expression analysis, our study generated the significant genes, ASPM, CDCA8, CENP5, CEP55, and PLK1 that may be biomarkers and has potential for treatment in HIV‐infected lung cancer.

### AUTHOR CONTRIBUTION

Study design: Yanzheng Song and Lin Wang. Data collection: Lin Wang, Liwei Wu, Zilu Wen and Laiyi Wan. Data analysis: Liwei Wu, Yongfang Chen and Laiyi Wan. Writing: Liwei Wu, Yongfang Chen and Laiyi Wan. Providing patients: Rong Liu and Leilei Li.

## FUNDING INFORMATION

This research was supported by a grant from the Shanghai Public Health Clinical Center fund (grant no. KY‐GW‐2021‐49).

## CONFLICT OF INTEREST

None of the authors has any conflict of interests.

## ETHICS STATEMENT

The Permission of the Hospital Ethics Committee was obtained by the Shanghai public health clinical center's ethics committee before operation for each patient. All authors confirm that all methods were performed in accordance with the relevant guidelines and regulations.

## Data Availability

All data included in this study will be obtained by contacting the corresponding author.
